# Seasonal patterns of *Schistosoma mansoni* infection within *Biomphalaria* snails at the Ugandan shorelines of Lake Albert and Lake Victoria

**DOI:** 10.1371/journal.pntd.0011506

**Published:** 2023-08-14

**Authors:** Peter S. Andrus, J. Russell Stothard, Christopher M. Wade

**Affiliations:** 1 School of Life Sciences, University of Nottingham, Nottingham, United Kingdom; 2 Department of Tropical Disease Biology, Liverpool School of Tropical Medicine, Pembroke Place, Liverpool, United Kingdom; George Washington University, UNITED STATES

## Abstract

Intestinal schistosomiasis is hyperendemic in many sub-Saharan African countries. In Uganda, it is endemic at both Lake Albert (LA) and Lake Victoria (LV) and caused by *S*. *mansoni* that uses *Biomphalaria* snails as obligatory intermediate snail hosts. To shed light on local patterns of infection, we utilised two PCR-based methods to detect *S*. *mansoni* within *Biomphalaria* spp. as collected at the Ugandan shorelines of Lake Albert and Lake Victoria from 2009–2010. Overall, at our Lake Albert sites, the mean infection prevalence was 12.5% (15 of 120 snails), while at our Lake Victoria sites the prevalence was 5% (3 of 60 snails). At our Lake Albert sites, the highest infection prevalence of 13.3% (8 of 60 snails) was at Walukuba, while at our Lake Victoria sites, the highest infection prevalence of 10% (2 of 20 snails) was at Lwanika. Three species of *Biomphalaria*, *B*. *pfeifferi*, *B*. *stanleyi* and *B*. *sudanica*, were identified at our Lake Albert collection sites, while only a single species, *B*. *choanomphala*, was identified at our Lake Victoria collection sites. *Biomphalaria stanleyi* (2 of 20 snails; 15%) had the highest infection prevalence, followed by *B*. *sudanica* (5 of 60 snails; 13.3%), *B*. *pfeifferi* (4 of 40 snails; 10%) and *B*. *choanomphala* (3 of 60 snails; 5%). Of the *Biomphalaria* species identified, *B*. *choanomphala* had the highest haplotype (gene) diversity score, followed by *B*. *stanleyi*, *B*. *sudanica* and *B*. *pfeifferi*. Sites with a higher mean prevalence of *S*. *mansoni* infection had higher intra-species haplotype diversity scores than sites with a lower mean prevalence. The wet seasons (LA: 13.3%; LV: 8.7%) had a consistently higher mean infection prevalence of *S*. *mansoni* than the dry seasons (LA: 9.5%; LV: 5%) for all species and all sites tested at both Lake Albert (n = 480) and Lake Victoria (n = 320), though the difference was not statistically significant.

## Author summary

Human schistosomiasis is a parasitic disease caused by the intravascular trematode genus *Schistosoma*. The disease is contracted through contact with contaminated freshwater sources infested with snails, which serve as the intermediate host for the parasite’s larval form. *Schistosoma mansoni* causes the intestinal form of the disease and utilises *Biomphalaria* as its intermediate snail host. To better understand the prevalence of *S*. *mansoni* infection, molecular detection methods can be used to monitor the levels and patterns of infection within *Biomphalaria* populations. In this study, the authors examined the prevalence of *S*. *mansoni* infection within *Biomphalaria* snails collected from six sites along the Ugandan shorelines of Lake Albert and Lake Victoria from 2009 to 2010. The study revealed that infection was more prevalent at Lake Albert compared to Lake Victoria. Different species of *Biomphalaria* snails were found at each lake, with some species having a higher infection prevalence than others. The study also investigated the impact of seasonality on infection prevalence, with the wet seasons having an overall higher prevalence of infection compared to the dry seasons, although the difference was not statistically significant. This research enhances our understanding of *S*. *mansoni* infection patterns among African *Biomphalaria* snails.

## Introduction

Schistosomiasis is a parasitic disease caused by infection with digenetic trematodes of the genus Schistosoma. It is estimated that 133 million children and 108 million adults are infected with schistosomiasis worldwide, with over 700 million people being at risk of infection [1]. Schistosomiasis is most prevalent in sub-Saharan Africa, with approximately 93% of infections and up to 90% of individuals at risk of infection living within sub-Saharan African countries [2,3]. The disease manifests as either intestinal (caused by *Schistosoma mansoni*, *S*. *intercalatum*, *S*. *japonicum* or *S*. *mekongi*) or urogenital forms (caused by *S*. *haematobium*) [4]. *Schistosoma mansoni* is the leading global cause of intestinal schistosomiasis in humans and accounts for 33% of all schistosomiasis cases [5].

Schistosomiasis is particularly prevalent in East Africa, with Tanzania having the highest national prevalence with 51.5% (an estimated 23.2 million infected) [6], followed by Uganda with 25.6% (11 million infected) [7] and Kenya with 14.5% (6 million infected) [8,9]. The distribution of schistosomiasis is dependent on the ecological requirements of the intermediate snail host, with the availability of freshwater habitats limiting the spread of schistosomiasis [10,11]. East Africa has a high prevalence of schistosomiasis due to the abundance of diverse freshwater environments (lakes, ponds, streams, dams and irrigation canals) that intermediate snail hosts inhabit [12]. Combined with poor water hygiene and sanitation, this provides an optimal environment for the transmission of schistosomiasis [12]. Sousa-Figueiredo et al. 2010 reported intestinal schistosomiasis is high among Ugandan shoreline villages, with Lake Albert having a prevalence of 82.2% in mothers and 68.7% in children, while Lake Victoria had a lower prevalence of infection, with 66.7% of mothers and 58.6% of children being infected [13]. This disparity in prevalence has been suggested to be the result of different species of *Biomphalaria* being present at each lake, with Lake Albert having reports of *B*. *pfeifferi*, *B*. *stanleyi* and *B*. *sudanica*, while Lake Victoria has had reports of *B*. *choanomphala*, *B*. *pfeifferi* and *B*. *sudanica* [14,15].

The freshwater snail genus *Biomphalaria* acts as the intermediate host for *S*. *mansoni*, with the African Great Lakes, Lake Albert and Lake Victoria providing a favourable habitat for multiple species of *Biomphalaria* [10,11,16,17]. All African *Biomphalaria* species are capable of transmitting *S*. *mansoni* infection [14], though some species (e.g. *B*. *pfeifferi*) are considered more important than others [18,19]. The rate of schistosome infection within a *Biomphalaria* population has traditionally been measured by observing how many snails shed cercariae over a 35–42 day period [20]. Previous studies using this traditional cercarial shedding method have shown that snails at Lake Albert consistently have a higher infection rate than snails at Lake Victoria [15,21]. Of the *Biomphalaria* species found at the African Great Lakes, *B*. *stanleyi* is reported as consistently having a high prevalence of *S*. *mansoni* infection [12,15,21,22], while *B*. *choanomphala* is reported as consistently having a low infection prevalence [15,21,23]. A meta-analysis by Hailegebriel et al. (2020) estimated the pooled prevalence of *S*. *mansoni* infection in *Biomphalaria* snails across Africa was on average 5.6% [19]. However, of the 51 studies investigating schistosome infection within intermediate snail hosts, only seven used molecular detection methods, while the rest used the traditional cercarial shedding method [19]. Molecular detection methods (molecular xenomonitoring) have several advantages over traditional cercarial shedding methods, as they do not require live snail specimens, are considerably less time consuming, can specifically detect *S*. *mansoni* infection, and can detect infection in both prepatent and shedding snails [24–31]. However, not all prepatent snails go on to shed cercariae, which can lead to exaggerated levels of infection when using molecular detection methods. Therefore, the use of both detection methods would ultimately give the best representation of infection prevalence within a snail population.

The prevalence of *S*. *mansoni* infection within a *Biomphalaria* population is affected by multiple factors. For example, past studies have associated snail populations with low levels of genetic variability with a higher prevalence of *S*. *mansoni* [32,33]. In addition, environmental factors such as altitude, water conductivity, water depth, water pH, temperature, droughts and floods have been shown to affect the prevalence of schistosome infections in snail populations [34–39]. As a result, many have speculated that *S*. *mansoni* prevalence differs throughout the year due to changes in environmental conditions between the seasons. For example, Uganda has a bimodal climate with two wet seasons (from March to May and from September to November) and two dry seasons (from December to February and from June to August) that take place every year [40]. Adoka et al. (2014) [41] reported that people living at the shoreline of Lake Victoria believed that intestinal schistosomiasis was more prevalent in the wet seasons than the dry seasons. Rowel et al. (2015) [15] found evidence in support of this, with their results showing that the number of *Biomphalaria* shedding cercariae was higher during the wet seasons than the dry seasons. However, there are few studies which explore the effect seasonality has on schistosome prevalence within snail populations [42].

Here we use two PCR-based, molecular xenomonitoring detection methods to investigate the infection prevalence of *S*. *mansoni* in *Biomphalaria* species found at the Ugandan shorelines of three Lake Albert and three Lake Victoria collection sites. Additionally, we investigate the effect seasonality has on the prevalence of *S*. *mansoni* infection by comparing the number of infected snails for each of the four wet and four dry seasons that took place between 2009 and 2010. Lastly, we measured the extent of the intraspecies genetic diversity present in the *Biomphalaria* species identified at the sites investigated, in order to determine whether there is any relationship between the prevalence of infection and the amount of snail host diversity.

## Materials and methods

### Sample sites and sample selection

**Fig 1:**
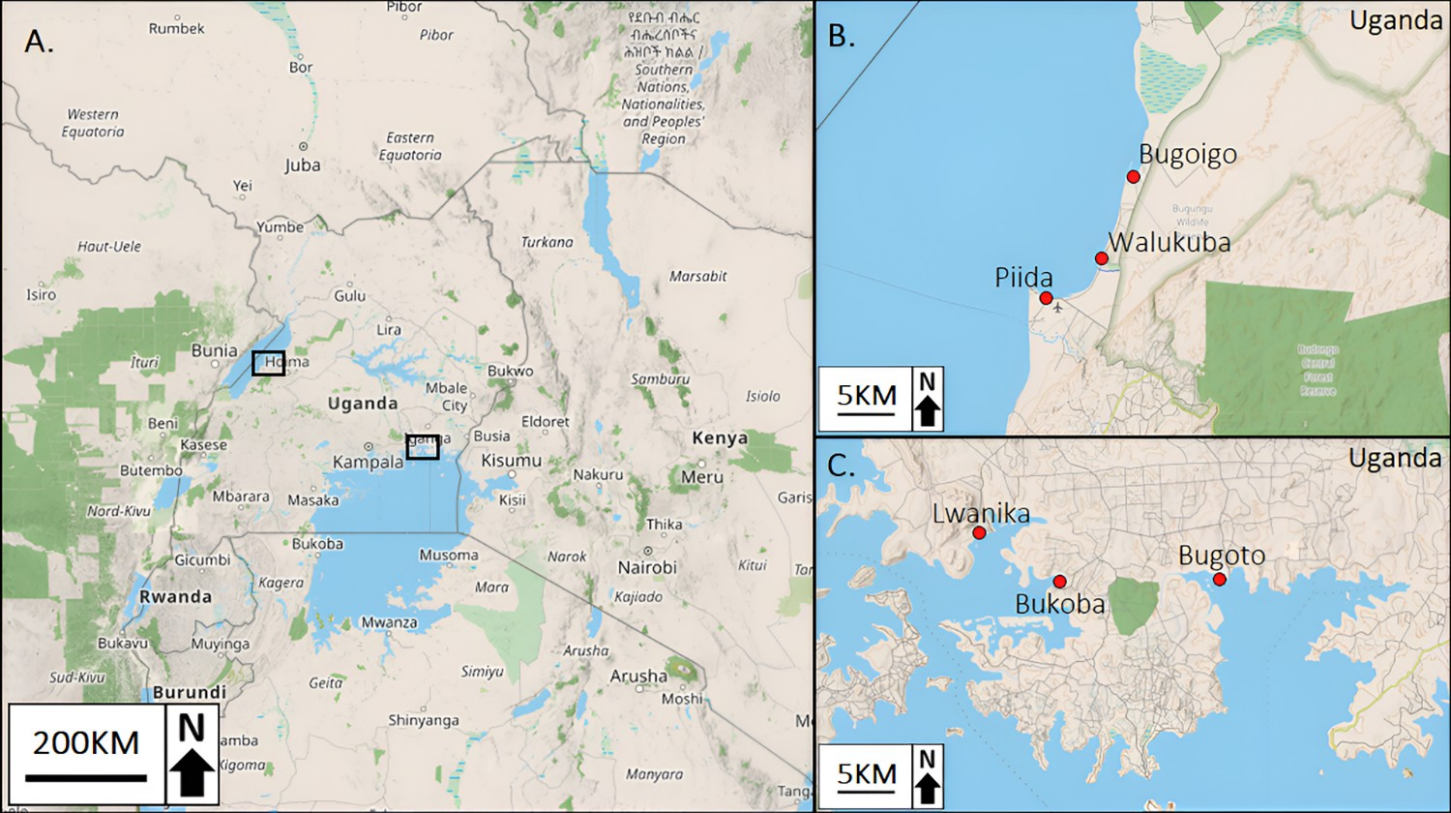
(A) Map showing the collection site locations at Lake Albert and Lake Victoria in Uganda. (B) The three collection sites of Lake Albert (Bugoigo, Piida and Walukuba) and (C) the three collection sites of Lake Victoria (Bugoto, Bukoba and Lwanika; Created using OpenStreetMap, https://www.openstreetmap.org).

*Biomphalaria* snails used in this study were collected once a month for 29 consecutive months between 2009 and 2010 from three sites in the Buliisa district on the Ugandan shoreline of Lake Albert (Bugoigo: 1.908°N, 31.409°E; Piida: 1.819°N, 31.328°E; Walukuba: 1.842°N, 31.378°E) and three sites in the Mayuge district on the Ugandan shoreline of Lake Victoria (Bugoto: 0.319°N, 33.620°E; Bukoba: 0.312°N, 33.492°E; Lwanika: 0.351°N, 33.446°E), as part of the Wellcome Trust funded, **S**chistosomiasis **I**n **M**others and **I**nfants (SIMI) project [13,43,44,45]. (Fig 1 and see Rowel et al., 2015 [15] for further details about the collections). At each site, snails were collected from both the lake edge, which was often marshy shoreline, and the deeper waters of the lake (~1m depth). Approximately half of the snails collected were preserved in 70% ethanol and were held as a reference archival collection at the Liverpool School of Tropical Medicine, UK. Overall, 2,645 randomly selected snails were preserved from the original 6,183 collected at Lake Albert, and 6,382 randomly selected snails were preserved from the original 13,172 collected at Lake Victoria.

### Snail identification and genetic diversity

All of the preserved *Biomphalaria* species collected over the two year period were initially identified to the species level using conchological identification methods [14]. At each site, twenty individuals of each species identified were selected for further molecular analysis. These selected individuals all came from the August 2010 collection, as this period had the highest number of viable specimens available. For each snail, DNA was extracted using a modified CTAB extraction method as described in Joof et al. (2020) [46], with extracted samples being resuspended in 100μl of TE, pH 8.0 (10mM Tris-HCl, 0.1mM EDTA) buffer. After extraction, DNA yields were measured using a NanoPhotometer N50 (Implen, München, Germany). The identification of each specimen was confirmed using 16S and COI genotyping. For the 16S gene, we used a modified version of the 16Sar/16Sbr primers designed by Palumbi et al. (1991) [47] (16Sarm: 5’-CTT CTC GAC TGT TTA TCA AAA ACA-‘3 and 16Sbrm: 5’-GCC GGT CTG AAC TCA GAT CAT-‘3). For COI, we used the universal COI primers designed by Folmer et al. (1994) [48] (LCO1490: 5’-GGT CAA ATC ATA AAG ATA TTG G-‘3 and HCO2198: 5’-TAA ACT TCA GGG TGA CCA AAA AAT CA-‘3). All PCR reactions were performed using Promega GoTaq G2 Master Mix buffer, with 1μl of DNA template added to 24μl of 1X Master Mix buffer (1U TAQ, 0.2μM primers, 200μMdNTPs, 3mM MgCl_2_). The PCR cycling conditions used for both the 16S and COI primer sets were identical, with an initial denaturation at 96°C for 1minute, followed by 34 cycles of 94°C for 1min, 50°C for 1min, 72°C for 1min and a final extension at 72°C for 10mins. All PCR products were electrophoresed on a 2% agarose gel containing ethidium bromide and were observed under UV light. All 16S and COI PCR products were purified and sequenced using Macrogen’s EZ-Seq service.

All sequences were aligned using the Muscle algorithm in the program Seaview v5 [49], with misaligned sections of the 16S and the COI being fixed by hand and sites for tree building selected using the Gblocks program [50]. Samples were identified to the species-level using a concatenated 16S and COI phylogenetic tree incorporating GenBank references from Jørgensen et al. (2007) [51], Plam et al. (2008) [52], Standley et al. (2014) [53] and Zhang et al. (2018) [54] (S1 Table). Phylogenetic trees were constructed using the Maximum Likelihood method, using a General Time Reversible model incorporating gamma correction (GTR+Γ) in the program PhyML v3.1 [55], with bootstrap analysis undertaken using 1000 replicates. After confirming which species were present at our Lake Albert and Lake Victoria sites, we then measured the genetic variability of each species using DNASP v6 [56] to calculate Haplotype (gene) diversity (Hd) scores and nucleotide diversity (π) values [57]. MEGA-X [58] was used to calculate pairwise distances, with distances corrected using the Maximum Composite Likelihood (MCL) method. Genealogical relationships of the 16S and COI haplotypes were constructed using Median-Joining (MJ) networks [59] using the software NETWORK v5 (Fluxus Technology Ltd. www.Fluxus-engineering.com; S2 Table).

### Infection detection

The prevalence of *S*. *mansoni* infection at Bugoigo, Piida, Walukuba, Bugoto, Bukoba and Lwanika was measured by initially testing twenty individuals of each species at a single time-point (August 2010). All of the DNA extracts were first tested using the LSU1iii/LSU3iii primers (LSU-1iii: 5′-TGC GAG AAT TAA TGT GAA TTG C-3′ and LSU-3iii: 5′- ACG GTA CTT GTC CGC TAT CG-3′) developed by Fontanilla et al. (2017) [60] to ensure that our DNA extracts were amplifiable. All PCR reactions were performed using Promega GoTaq G2 Master Mix buffer, with 1μl of DNA template added to 24μl of 1X Master Mix buffer (1U TAQ, 0.2μM primers, 200μM dNTPs, 3mM MgCl_2_). The PCR cycling conditions for the LSU-1iii/3iii primers was an initial denaturation at 96°C for 2min, followed by 35 cycles of 94°C for 30sec, 45°C for 1 min, 72°C for 2min and a final extension step at 72°C for 5 min.

After confirming the quality of our DNA extracts, we tested for *S*. *mansoni* infection using two different primer sets, firstly Sm^F/R^ (designed by Sandoval et al. 2006) [27] and then ND5 (designed by Lu et al. 2016) [30]. Only samples that tested positive with the Sm^F/R^ primer set were subjected to further testing using the ND5 primer set. This additional testing was carried out because the ND5 primer set possesses the ability to differentiate between human and non-human schistosome species based on the length of the diagnostic band [30]. All PCR reactions were performed using Promega GoTaq G2 Master Mix buffer, with 1μl of DNA template diluted to 50ng/μl. Alongside the *Biomphalaria* samples, two negative controls (water and uninfected *B*. *glabrata* DNA) and two positive controls (pure *S*. *mansoni* DNA and infected *B*. *glabrata* DNA) were also included. These controls were provided by Professor Mike Doenhoff, School of Biology, University of Nottingham. The PCR reaction mixture and cycling conditions for the Sm^F/R^ and ND5 primer sets were followed precisely as described by Sandoval et al. (2006) [27] and Lu et al. (2016) [30], respectively. *Schistosoma mansoni* infection was confirmed by running the PCR products on a 2% agarose gel containing ethidium bromide and observing whether a diagnostic band was present under UV light.

To examine the seasonal prevalence of infection at each site and of each species, we tested twenty individuals at both Lake Albert and Lake Victoria for each of the four wet (March to May and September to November) and four dry (December to February and June to August) seasons that occurred within the two year collection period (January 2009 to December 2010; rainfall data for Uganda is provided in S1 Fig). However, due to the limited number of samples available at Piida and Bukoba, only two of the three Lake Albert (Bugoigo Walukuba) and Lake Victoria (Bugoto and Lwanika) sites could be tested. Likewise, due to the limited number of *B*. *stanleyi* samples available, only *B*. *choanomphala* (Bukoba and Lwanika), *B*. *pfeifferi* (Walukuba) and *B*. *sudanica* (Walukuba and Bugoigo) could be tested. SPSS v26 (IBM, Armonk, USA) [61] was used to perform a Pearson’s chi-squared (X^2^) test with Yates’ correction to compare the prevalence of infection. The summary of the samples tested for infection can be found in S3 Table.

### GenBank accessions

GenBank accession numbers for the *Biomphalaria* 16S and COI sequences used from Jørgensen et al. (2007) [51], Plam et al., 2008 [52], Standley et al. (2014) [53] and Zhang et al. (2018) [54] can be found in S1 Table. The DNA sequences generated in this study are available in GenBank accession numbers OQ924749-OQ924929 for the 16S gene and OQ849817-OQ849997 for the COI gene (further information can be found in S1 and S2 Tables).

## Results

### Infection prevalence at the African great Lakes

Of the sites tested, Lake Albert had the highest infection prevalence of *S*. *mansoni*, with an overall prevalence of 12.5% (15 PCR positive snails out of 120). Conversely, our Lake Victoria sites had a lower prevalence of only 5% (3/60). When partitioned by site, the Lake Albert sites had a higher mean prevalence of infection than the Lake Victoria sites (Table 1). Walukuba had the highest prevalence of infection of the Lake Albert sites with 13.3% (8/60), followed by Bugoigo with 12.5% (5/40) and Piida with 10% (2/20) (Table 1). Of the Lake Victoria sites, Lwanika had the highest prevalence with 10% (2/20), followed by Bugoto and Bukoba with 5% (1/20) for both sites (Table 1). All of our Sm^F/R^ positive *Biomphalaria* samples were confirmed to be infected with *S*. *mansoni* as every positive sample gave a diagnostic band length of ~302bp when tested with the ND5 primer set.

**Table 1:** Mean prevalence of *S*. *mansoni* infection and the number of unique 16S/COI haplotypes (No.), haplotype diversity scores (Hd) and nucleotide diversity values (π) of each *Biomphalaria* species genotyped at our Lake Albert and Lake Victoria collection sites (August 2010 collection).

Lake Albert									
	Species	No. Infected	Site Infection	16S			COI		
				No.	Hd	π	No.	Hd	π
Bugoigo	*B*. *sudanica* (*n =* 20)	3	12.5%	7	0.784	0.000	3	0.532	0.002
	*B*. *pfeifferi* (*n =* 20)	2		2	0.337	0.000	4	0.489	0.001
Piida	*B*. *sudanica* (*n =* 20)	2	10%	6	0.716	0.000	2	0.521	0.002
Walukuba	*B*. *stanleyi* (*n =* 20)	3	13.3%	10	0.884	0.002	10	0.815	0.003
	*B*. *sudanica* (*n =* 20)	3		10	0.884	0.001	4	0.553	0.002
	*B*. *pfeifferi* (*n =* 20)	2		3	0.468	0.001	6	0.832	0.002
Lake Victoria									
	Species	No. Infected	Site Infection	16S			COI		
				No.	Hd	π	No.	Hd	π
Bugoto	*B*. *choanomphala* (*n =* 20)	1	5%	11	0.884	0.008	5	0.774	0.004
Bukoba	*B*. *choanomphala* (*n =* 20)	1	5%	15	0.958	0.007	10	0.89	0.005
Lwanika	*B*. *choanomphala* (*n =* 20)	2	10%	16	0.963	0.008	9	0.826	0.005

Note: *Schistosoma mansoni* infection was determined based on whether snails had a diagnostic band for both Sm^F/R^ (~350bp) and ND5 (~302bp).

Of the sites tested, we found three *Biomphalaria* species, *B*. *pfeifferi*, *B*. *stanleyi* and *B*. *sudanica*, at Lake Albert and one species, *B*. *choanomphala*, at Lake Victoria (S2 and S3 Figs). Of the four species identified, *B*. *stanleyi* had the highest prevalence of *S*. *mansoni* infection with 15% (3/20), followed by *B*. *sudanica* with 13.3% (8/60), *B*. *pfeifferi* with 10% (4/40), and *B*. *choanomphala* with 5% (3/60) (Table 1). *Biomphalaria choanomphala* exhibited two different shell morphologies, with 45 of the 60 snails exhibiting a lacustrine shell morphology (S2 Fig); all of the infected *B*. *choanomphala* snails at our Lake Victoria sites exhibited the lacustrine shell morphology. In addition to the four *Biomphalaria* species, we identified an Asian *Gyraulus* species at both Lake Albert and Lake Victoria (S2 Fig). There have been no published reports of *Schistosoma* infection in *Gyraulus*, and we detected no cases of *S*. *mansoni* infection in the Asian *Gyraulus* species found at Lake Albert (0/10) or Lake Victoria (0/10).

### Genetic diversity of the *Biomphalaria* species at the African great Lakes

**Fig 2:**
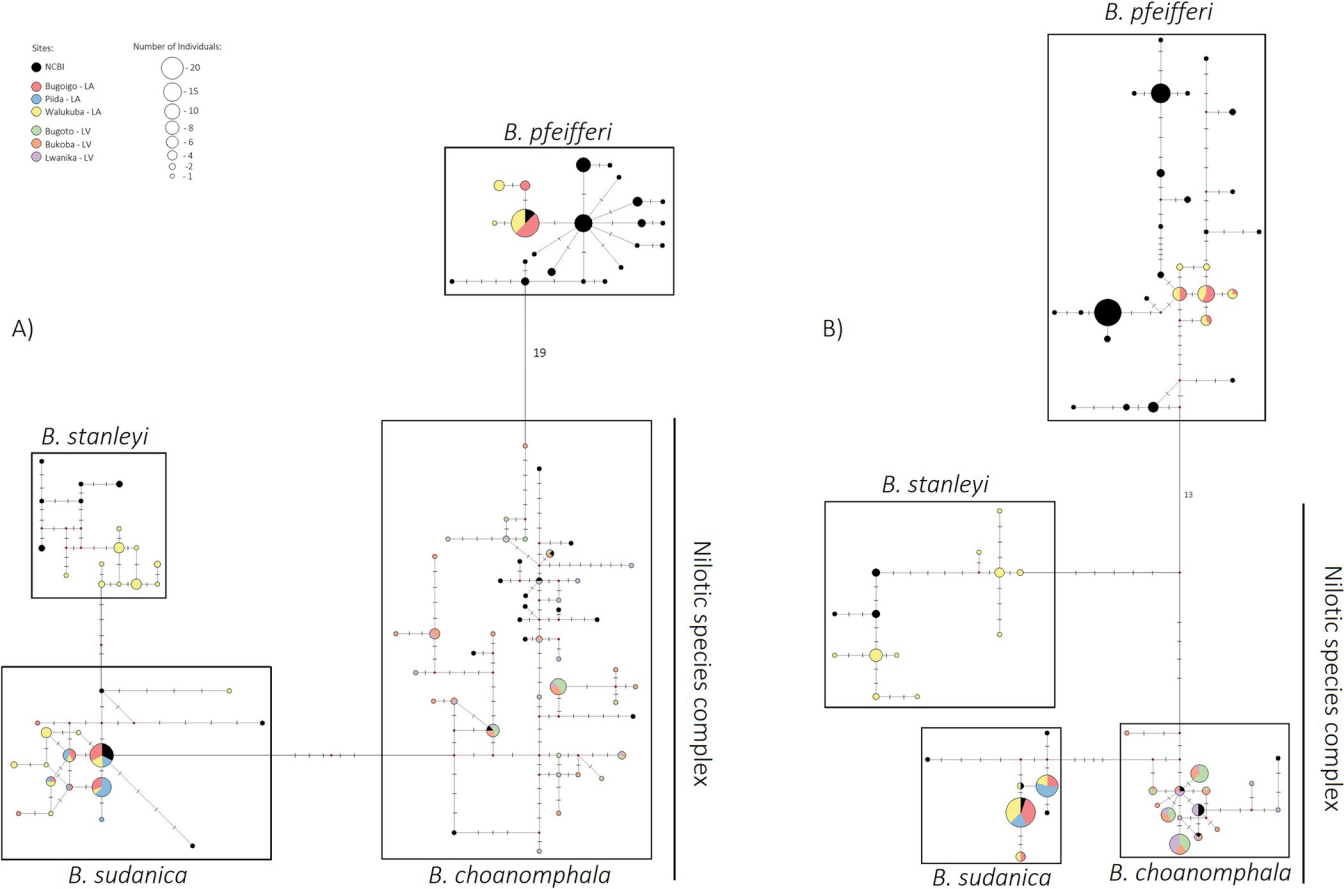
Median-Joining haplotype network of the *Biomphalaria* species found at Lake Albert (*B*. *pfeifferi n =* 40; *B*. *stanleyi n =* 20; *B*. *sudanica n =* 60) and Lake Victoria (*B*. *choanomphala n =* 60) using (A) 16S rRNA gene fragment (395bp) and (B) Cytochrome Oxidase Subunit I gene fragment (520bp). This network was generated using the software NETWORK v5. Circles represent each haplotype and circle size represents the numbers of individuals sharing a haplotype. Diamonds represent intermediate haplotypes, while hatch marks between points represent the number of nucleotide substitutions (substitutions more than five are indicated by numbers). Gaps were included in the 16S and COI alignments. Reference sequence information for the 16S and COI networks can be found in S1 and S2 Tables, respectively.

Of the *Biomphalaria* species found at our collection sites, *B*. *choanomphala* (*n =* 60) had 31 haplotypes for the 16S gene fragment, followed by *B*. *sudanica* (*n =* 60) with 14, *B*. *stanleyi* (*n =* 20) with 10 and *B*. *pfeifferi* (*n =* 40) with four (Table 1 and Fig 2A). For the COI gene fragment, *B*. *choanomphala* had 14 haplotypes, followed by *B*. *stanleyi* with 10, *B*. *pfeifferi* with six and *B*. *sudanica* with four (Table 1 and Fig 2B). Of the *B*. *choanomphala* snails sequenced, the lacustrine specimens had 21 unique 16S haplotypes and 12 unique COI haplotypes, while the non-lacustrine specimens had 14 unique 16S haplotypes and 8 unique COI haplotypes. Several of the 16S and COI haplotypes were shared between lacustrine and non-lacustrine individuals (S4 Fig). The haplotype diversity (Hd) scores for the 16S gene were highest for *B*. *choanomphala* with 0.945, followed by *B*. *sudanica* with 0.833, *B*. *stanleyi* with 0.884 and *B*. *pfeifferi* with 0.422. For the COI gene, haplotype diversity (Hd) scores were highest for *B*. *choanomphala* with 0.842, followed by *B*. *stanleyi* with 0.815, *B*. *pfeifferi* with 0.618 and *B*. *sudanica* with 0.553. Overall, the haplotypes were not highly divergent for both the 16S and COI. The nucleotide diversity values were highest for the *B*. *choanomphala* populations at Lake Victoria for both the 16S (0.007–0.008) and COI (0.004–0.005), while all of the *Biomphalaria* species at Lake Albert had very low nucleotide diversity values for both the 16S (0.000–0.002) and COI (0.001–0.003; Table 1). The intra-species pairwise distances of the 16S was the highest for *B*. *choanomphala* (0.0–1.8%), followed by *B*. *stanleyi* (0.0–0.8%), *B*. *sudanica* (0.0–0.8%) and *B*. *pfeifferi* (0.0–0.1%). Conversely, the intra-species pairwise distances of the COI was the highest for *B*. *pfeifferi* (0.0–1.4%), followed by *B*. *stanleyi* (0.0–1.3%), *B*. *choanomphala* (0.0–1.2%) and *B*. *sudanica* (0.0–0.4%).

### Seasonality of infection prevalence

At Lake Albert we examined the seasonal changes in infection prevalence at two sites (Bugoigo and Walukuba). One species (*B*. *sudanica*) was tested at Bugoigo, while two species (*B*. *pfeifferi* and *B*. *sudanica*) were tested at Walukuba. Piida and *B*. *stanleyi* were not tested due to a lack of samples. At Bugoigo, the wet seasons had a mean infection prevalence of 12.5% (10/80), while the dry seasons had a mean infection prevalence of 10% (8/80) (Table 2). At Walukuba, the wet seasons had a mean infection prevalence of 13.8% (22/160), while the dry seasons had a mean infection prevalence of 9.4% (15/160) (Table 2).

**Table 2:** Mean prevalence of infection of the wet and dry seasons at Lake Albert and Lake Victoria between 2009–2010.

Lake Albert						
Site	Species	First Dry (*n =* 40)	First Wet (*n =* 40)	Second Dry (*n =* 40)	Second Wet (*n =* 40)	Overall Infection (*n =* 160)
Walukuba	*B*. *pfeifferi*	4 (10%)	6 (15%)	4 (10%)	6 (15%)	20 (12.5%)
	*B*. *sudanica*	3 (7.5%)	5 (12.5%)	4 (10%)	5 (12.5%)	17 (10.6%)
Bugoigo	*B*. *sudanica*	4 (10%)	5 (12.5%)	4 (10%)	5 (12.5%)	18 (11.3%)
Lake Victoria						
Site	Species	First Dry (*n =* 40)	First Wet (*n =* 40)	Second Dry (*n =* 40)	Second Wet (*n =* 40)	Overall Infection (*n =* 160)
Lwanika	*B*. *choanomphala*	2 (5%)	3 (7.5%)	3 (7.5%)	4 (10%)	12 (7.5%)
Bugoto	*B*. *choanomphala*	1 (2.5%)	3 (7.5%)	2 (5%)	4 (10%)	10 (6.3%)

Note: First Dry: Dec-Feb; First Wet: Mar-May; Second Dry: Jun-Aug; Second Wet: Sep-Nov.

At Lake Victoria, we examined the seasonal changes in prevalence of infection among *Biomphalaria* populations (*B*. *choanomphala*) at two sites (Bugoto and Lwanika). Bukoba was not tested due to a lack of samples. At Lwanika, the wet seasons had a mean infection prevalence of 8.8% (7/80), while the dry seasons had a mean infection prevalence of 6.3% (5/80) (Table 2). Bugoto had a mean infection prevalence of 8.8% (7/80) for the wet seasons and 3.8% (3/80) for the dry seasons (Table 2).

Overall, the prevalence of *S*. *mansoni* infection was consistently higher in the wet seasons than the dry seasons for both Lake Albert and Lake Victoria (Table 2 and S1 Fig). The overall mean prevalence of infection at Lake Albert for the four wet seasons was 13.3% (32/240), while the four dry seasons was 9.5% (23/240) (Table 2). Similarly, the overall mean prevalence of infection at Lake Victoria was 8.7% (14/160) for the wet seasons and 5% (8/160) for the dry seasons (Table 2). Nevertheless, a chi-square (X^2^) analysis found there was no significant difference in the prevalence of infection between the wet and dry seasons (*p* = 0.252 for Lake Albert and *p* = 0.269 for Lake Victoria). When comparing the prevalence of infection for the first and second wet season we found no difference for the Lake Albert sites. Likewise, there was no difference in infection prevalence for the first and second dry season. For Lake Victoria, we found that the first wet season had a lower mean prevalence of infection than the second wet season. Similarly, the first dry season also had a lower prevalence of infection than the second dry season (Table 2).

In order to test consistency in our infection prevalence estimates, we compared the prevalence of infection measured in our seasonality dataset against our single time point (August 2010) dataset. The single time point dataset found a mean infection prevalence of 12.5% (15/120) for Lake Albert, while the seasonality dataset found a mean infection prevalence of 11.5% (55/480). Lake Victoria had an infection prevalence of 5% (3/60) for the single time point dataset, while the seasonality dataset had an infection prevalence of 7.2% (23/320). Of the species tested, *B*. *sudanica* had an infection prevalence of 13.3% for the single time point dataset and an infection prevalence of 10.9% for the seasonality dataset. The mean infection prevalence of the *B*. *pfeifferi* snails was 10% for the single time point dataset and 12.5% for the seasonality dataset. Lastly, the *B*. *choanomphala* snails had a mean infection prevalence of 5% for the single time point dataset and 6.9% for the seasonality dataset. A chi-square (X^2^) analysis found there was no significant (P> 0.05) difference in the prevalence of *S*. *mansoni* infection in *Biomphalaria* snails between the two datasets. The overall averages for both datasets can be found in S3 Table.

## Discussion

Of the six sites investigated which formed the surveillance area for the SIMI project, we found that Lake Albert (12.5%) had a higher prevalence of infected *Biomphalaria* snails than Lake Victoria (5%). Similarly, Rowel et al. (2015) [15] also reported that Lake Albert had a higher prevalence of shedding *Biomphalaria* snails (8.9%) compared to the *Biomphalaria* snails found at Lake Victoria (2.1%). When partitioned by site, we found Walukuba (13.8%) had the highest prevalence of infection of our Lake Albert sites, while Lwanika (10%) had the highest prevalence of infection of the Lake Victoria sites. Likewise, Rowel et al. (2015) [15] found that Walukuba (12.3%) had the highest prevalence of shedding *Biomphalaria* snails of the Lake Albert sites and Lwanika (3.8%) had the highest prevalence of shedding *Biomphalaria* snails of the Lake Victoria sites. Our result of Lake Albert having a higher prevalence of *S*. *mansoni* infection than Lake Victoria is consistent with previous findings [15,21,22,62]. The Vector Control Division (VCD) of the Ugandan Ministry of Health have had concerns about this issue, as despite the similar transmission rates of schistosomiasis and comparable mass drug administration programs present at both of the Great African Lakes, Lake Albert consistently has higher levels of severe morbidity compared to Lake Victoria. The **U**ganda **S**chistosomiasis **M**ultidisciplinary **R**esearch **C**entre (U-SMRC) suggests several hypotheses as to why there is a higher prevalence of schistosomiasis at the Ugandan shoreline of Lake Albert when compared to the Ugandan shoreline of Lake Victoria: (I) variations in the immune systems of the local people (e.g. differences in microbiome, nutrition and lifestyle); (II) the genetic makeup of the parasite populations (e.g. differences in immunogenic/ immunoregulatory antigens expressed by the parasite and varying levels of praziquantel resistance); (III) the abundance and number of snail species found near human activity (e.g. differences in susceptibility of the snail host and the intensity of exposure to the parasite) [63].

### Infection prevalence of the *Biomphalaria* species found at the African great Lakes

When partitioned by species, we found that *B*. *stanleyi* (15%) had the highest prevalence of infection at our Lake Albert sites, followed by *B*. *sudanica* (13.3%) and *B*. *pfeifferi* (10%). Our results are similar to Kazibwe et al. (2006) [12] and Rowel et al. (2015) [15], who similarly reported *B*. *stanleyi* snails as having the highest prevalence of infection at Lake Albert. At our Lake Victoria sites, *B*. *choanomphala* had an infection prevalence of 5%, with all of the infected individuals having a lacustrine shell morphology. Our results are similar to Mutuku et al. (2021) [64], who reported that *S*. *mansoni* infection and cercarial production was significantly higher in the lacustrine form of *B*. *choanomphala* than the non-lacustrine form, regardless of miracidium dosage or whether the eggs came from allopatric or sympatric sources. However, Rowel et al. (2015) [15] and Gouvras et al. (2017) [65] found the opposite trend, with the non-lacustrine form of *B*. *choanomphala* having a higher *S*. *mansoni* infection rate than the lacustrine form.

When compared to the original Rowel et al (2015) [15] study, our results observed a higher prevalence of *S*. *mansoni* infection at both the Lake Albert and Lake Victoria sites. Likely, this is a result of using molecular detection methods, which typically show higher levels of infection when compared to the traditional cercarial shedding method [30,46,66]. This is due to infected *Biomphalaria* snails not always producing cercariae during the usual 35–49 day incubation period. For example, colder temperatures can lead to delays in sporocyst development and shedding [34]. Similarly, delays to sporocyst development and shedding can arise due to the immune response to infection. The snail’s immunological response does not guarantee the complete eradication of all sporocysts and some sporocysts can release cercariae up to ten months post infection [67,68]. Ultimately these prepatent snails will be undetectable by the cercarial shedding method but are still detectable by molecular methods [30,46]. However, molecular methods can also overestimate the number of snails that present a risk. Lu et al. (2016) [30] found that not all PCR positive *Biomphalaria* snails went on to shed cercariae; some snails were able to successfully encapsulate and degrade the sporocysts during the prepatent period, which resulted in the infection failing. The chance of this happening was shown to be dependent on species, with the majority of PCR positive *B*. *pfeifferi* snails (60%) going on to shed cercariae, while only a minority of PCR positive *B*. *sudanica* snails (10%) went on to shed cercariae. It seems whether an infection is successful or not is dependent on schistosome-snail compatibility, with compatible schistosomes being able to successfully evade the host’s immune defences [69,70,71]. This means that a snail that is PCR positive for infection may not necessarily be capable of spreading that infection on to humans.

Moreover, Rowel et al. (2015) [15] reported that of the snails shedding cercariae, only 15.8% at Lake Albert and 13.9% at Lake Victoria were shedding *S*. *mansoni* cercariae (identified using general anatomical appearance) [72] as opposed to shedding cercariae of trematode species with no medical importance. Likely this difference in *S*. *mansoni* prevalence is the result of snails being co-infected with both *S*. *mansoni* and non-*S*. *mansoni* sporocysts simultaneously [66,73], which makes it more difficult to reliably identify the presence of *S*. *mansoni* cercariae since these *S*. *mansoni* cercariae can be obscured by other non-medically important cercariae and therefore missed. Molecular detection methods are able to detect more reliably whether or not *S*. *mansoni* is present, while ignoring the non-*S*. *mansoni* sporocysts.

### Infection prevalence and Host-Snail genetic diversity

We found that the *Biomphalaria* species found at our Lake Victoria sites (*B*. *choanomphala*) had higher intraspecies genetic diversity than the *Biomphalaria* species (*B*. *pfeifferi*, *B*. *stanleyi* and *B*. *sudanica*) found at our Lake Albert sites. Furthermore, our Lake Victoria sites had a lower prevalence of infection than our Lake Albert sites. This is consistent with previous studies that have reported higher levels of intra-species genetic variation in host snails being linked to a lower prevalence of infection [32,33]. However, when we examined each of the sites individually, we found that sites which had a higher prevalence of infection also had *Biomphalaria* populations with higher levels of intraspecific genetic diversity (Table 1). For example, when we compared the haplotype diversity scores of the 16S and COI genes for the *B*. *pfeifferi* snails found at Walukuba with the *B*. *pfeifferi* snails found at Bugoigo, we found Walukuba had both a higher amount of genetic diversity (16S Hd: 0.468; COI Hd: 0.832) and a higher prevalence of infection (13.3%) than Bugoigo (16S Hd: 0.337; COI Hd: 0.489; infection prevalence: 12.5%) (Table 1). Similarly, we also found this trend for both *B*. *sudanica* and *B*. *choanomphala* snails (Table 1). *B*. *sudanica* snails at Walukuba had both a higher amount of genetic diversity (16S Hd: 0.884; COI Hd: 0.553) and prevalence of infection (13.3%) than *B*. *sudanica* snails found at Bugoigo (16S Hd: 0.784; COI Hd: 0.532; infection prevalence: 12.5%) and Piida (16S Hd: 0.716; COI Hd: 0.521; infection prevalence: 10%) (Table 1). Likewise, the *B*. *choanomphala* snails at Lwanika had higher amounts of genetic diversity (16S Hd: 0.963; COI Hd: 0.826) and prevalence of infection (10%) than the *B*. *choanomphala* snails at Bugoto (16S Hd: 0.884; COI Hd: 0.774; infection prevalence: 5%) (Table 1).

*Biomphalaria* snails within a population have shown variability in their susceptibility to *S*. *mansoni* infection, with some individuals being successfully infected and others remaining resistant, resulting in a phenomenon known as "compatibility polymorphism". The underlying reason of why this occurs is not yet fully understood, but two hypotheses have been suggested to explain this phenomenon, the “resistance hypothesis” and the “matching hypothesis” [74]. The former suggests that the snail host’s resistance and susceptibility status play a significant role in determining whether infection is successful, as vulnerable individuals lack the ability to recognise the parasite upon entry or produce an effective immunological response in time [75]. Previous research has shown differences in immune-related genes between compatible and incompatible snails, supporting this hypothesis [76]. Conversely, the latter hypothesis proposes that the success or failure of an infection is not determined by the susceptibility or resistance of an individual, but rather by the level of compatibility between the host and parasite phenotypes, suggesting all snails are potentially susceptible to infection if they encounter a schistosome with a matching phenotype [77,78]. Previous experimental treatments have supported this hypothesis, by showing infection rates increase when the phenotypic diversity of miracidia increases [76]. Of the two hypotheses suggested, our results support the assertions proposed by the matching hypothesis, as we found the prevalence of infection increased alongside snail host genetic diversity. This possibly suggests that sites with more diverse snail host populations have a higher probability of the parasite encountering a compatible host, while sites with lower levels of snail host genetic diversity have a lower probability of the parasite finding a suitable host due to fewer possible combinations being available.

### Infection prevalence and seasonality

At our Lake Albert sites, we found that the wet seasons (March to May and September to November) had a higher prevalence of infection (13.3%) than the dry seasons (December to February and June to August) (9.6%). This was also the case at our Lake Victoria sites, with the wet seasons having a higher prevalence of infection (8.7%) than the dry seasons (5%). Rowel et al. (2015) [15] also observed a higher number of shedding *Biomphalaria* snails during the wet seasons at Lake Albert and Lake Victoria. Moreover, Kazibwe et al. (2006) [12] found the highest rates of cercarial shedding in *B*. *stanleyi* and *B*. *sudanica* snails at Lake Albert took place during the wet seasons. Similarly, Wolmarans et al. (2002) [79] found that South African *B*. *pfeifferi* snails collected during the wet season (January to April) had a higher cercarial shedding rate than *B*. *pfeifferi* snails collected during either the cold (May to August) or the warm (September to December) dry seasons. However, depending on where the parasitological survey is undertaken can lead to contradictory results as studies undertaken in Ethiopia [80], Nigeria [81], Tanzania [82] and Sudan [83] have found the opposite trend, with the dry seasons having a higher rate of *S*. *mansoni* infected snails than the wet seasons. Moreover, our chi-square (X^2^) analysis found that the prevalence of infection during the wet seasons was not significantly higher than the prevalence of infection during the dry seasons for both Lake Albert and Lake Victoria.

## Supporting information

S1 Fig The mean monthly rainfall at Lake Albert and Lake Victoria during the wet (March-May & September-November) and dry (December-February & June-August) seasons. Rainfall data was collected by weather stations located near Lake Albert (Erusi Forest, Ihungu, and Kiryanga Gombolola) between 1904 and 2001, and by weather stations near Lake Victoria (Gayaza Isingiro, Ntusi, and Entebbe) between 1900 and 2005. Data and figures were adapted from the Nile basin water resources atlas (Nile Basin Initiative, 2017) [40].
(TIF)

S2 Fig Shell morphologies of *the* preserved snails collected at the Ugandan shorelines of Lake Albert and Lake Victoria. *Biomphalaria pfeifferi*, *B*. *stanleyi* and *B*. *sudanica* were present at Lake Albert, while the two morphotypes (non-lacustrine and lacustrine) of *B*. *choanomphala* were present at Lake Victoria. In addition, an invasive, unidentified Asian *Gyraulus* species was present at Lake Albert and Lake Victoria. The shells are viewed from the apertural (left) and umbilical (right) shell angles.
(TIF)

S3 Fig Maximum likelihood tree of the Cytochrome C Oxidase Subunit I (COI; 500bp) gene fragment. This tree was generated using PhyML v3.1 using a GTR+Γ model and is rooted on *Biomphalaria glabrata*. Numbers on branches indicate the bootstrap percentages for 1000 replicates (bootstrap support values under 50% are not shown). The scale bar represents sequence divergence. Samples labelled ‘*cf*.*’* had shell morphologies’ that looked like a specific species but were identified by the original authors as another species using molecular methods.
(TIF)

S4 Fig Median-Joining haplotype network of the *B*. *choanomphala* (n = 60) snails found at our Lake Victoria sites using the 16S rRNA gene fragment (395bp) and Cytochrome oxidase subunit I gene fragment (520bp). Each of the *B*. *choanomphala* snails shown are colour-coded to indicate whether they exhibited a non- lacustrine (black) or lacustrine (white) shell morphology. This network was generated using the software NETWORK v5. Circles represent each haplotype and circle size represents the numbers of individuals sharing a haplotype. Diamonds represent intermediate haplotypes, while hatch marks between points represent the number of nucleotide substitutions (substitutions more than five are indicated by numbers). Gaps were included in the 16S and COI alignments.
(TIF)

S5 Fig Seasonal prevalence of *Schistosoma mansoni* infection at our Lake Albert (A-C) and Lake Victoria (D-E) sites over the course of two years (2009–2010). *Biomphalaria sudanica* (*n =* 320) was tested at two sites in Lake Albert (A: Bugoigo & B: Walukuba), while *B*. *pfeifferi* (*n =* 160) was tested at one site (C: Walukuba). *Biomphalaria choanomphala* (*n =* 320) was tested at two sites at Lake Victoria (D: Bugoto & E: Lwanika). Black bars indicate the percentage of infected individuals (*n =* 20). (Dry 1: January-February 2009; Wet 1: March-May 2009; Dry 2: June-August 2009; Wet 2: September-November 2009; Dry 3: December 2009-February 2010; Wet 3: March-May 2010; Dry 4: June-August 2010; Wet 4: September-November 2010).
(TIF)

S1 Table GenBank accession numbers and corresponding references for the 16S/COI phylogenetic tree. (DOCX)

S2 Table GenBank accession numbers for the 16S and COI haplotype network. (DOCX)

S3 Table Infection prevalence of the single time point dataset and the seasonality dataset for our Lake Albert and Lake Victoria sites. (DOCX)
