# Supplementary figures and images for: Seasonal patterns of Schistosoma mansoni infection within Biomphalaria snails at the Ugandan shorelines of Lake Albert and Lake Victoria
Source: PLoS Negl Trop Dis. 2023 Aug 14;17(8):e0011506. doi: 10.1371/journal.pntd.0011506 (PMC10424865; doi:10.1371/journal.pntd.0011506)

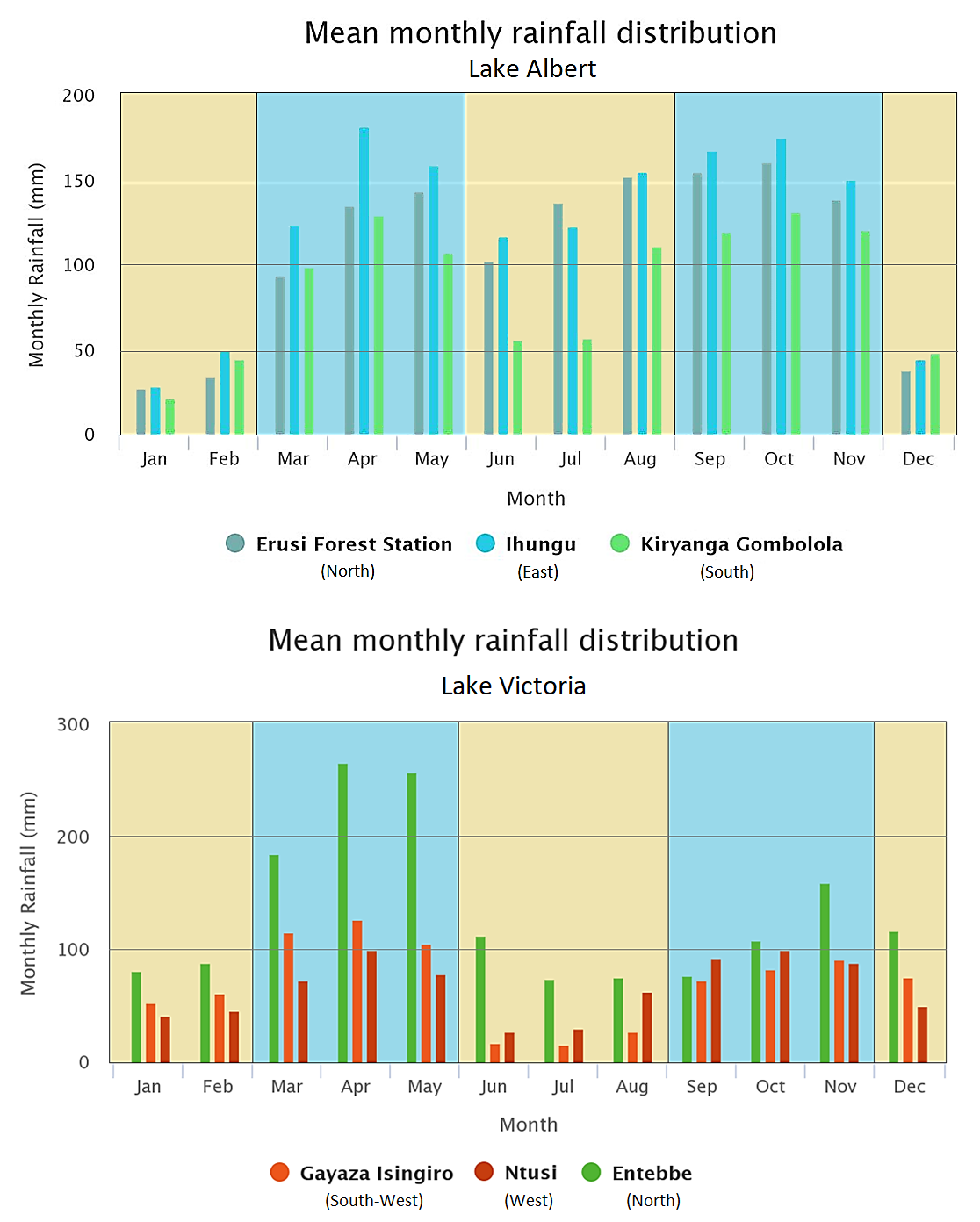

Supplement: S1 Fig — Rainfall data was collected by weather stations located near Lake Albert (Erusi Forest, Ihungu, and Kiryanga Gombolola) between 1904 and 2001, and by weather stations near Lake Victoria (Gayaza Isingiro, Ntusi, and Entebbe) between 1900 and 2005. Data and figures were adapted from the Nile basin water resources atlas (Nile Basin Initiative, 2017) [40]. (TIF) [file pntd.0011506.s001.tif]

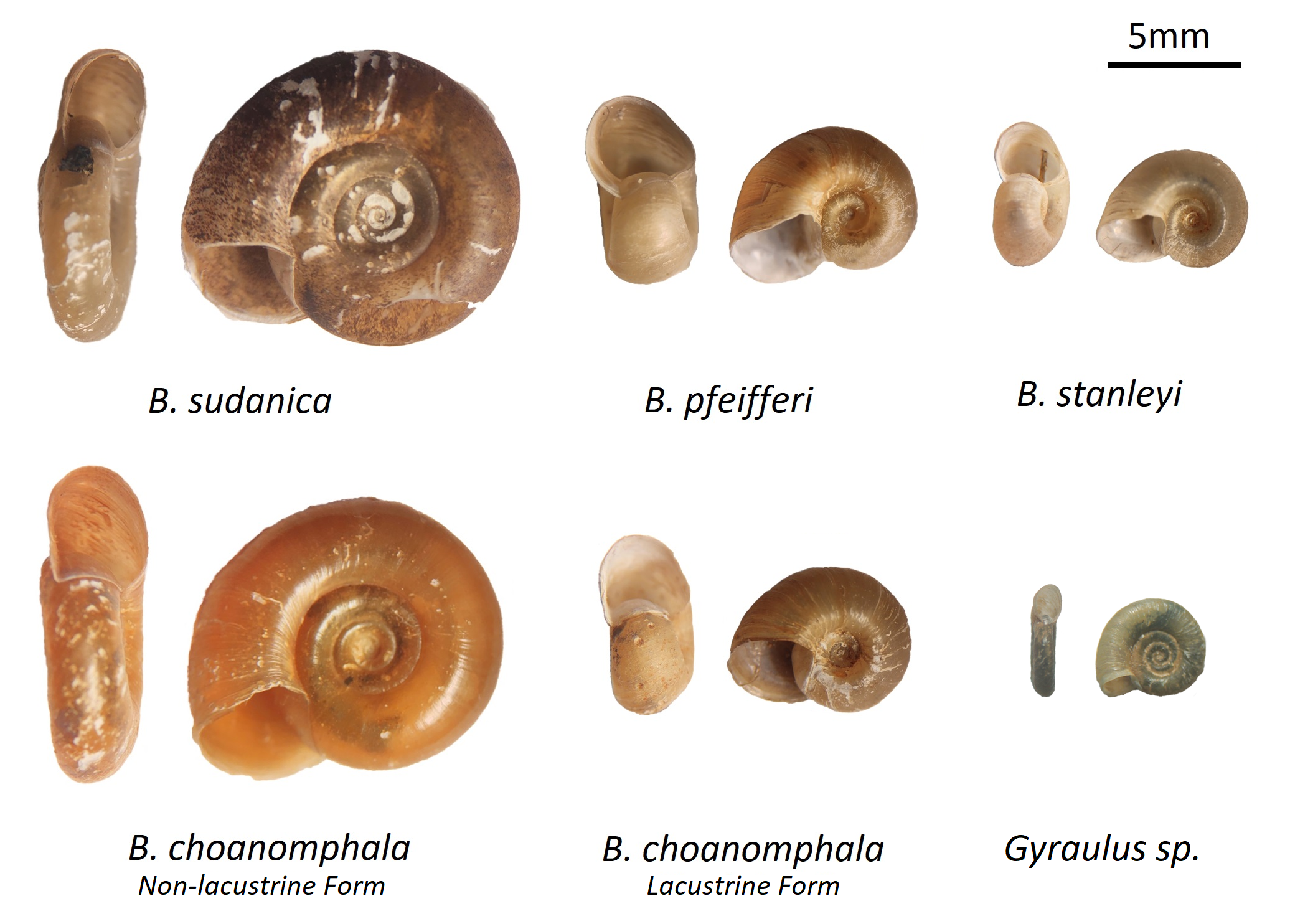

Supplement: S2 Fig — Biomphalaria pfeifferi, B. stanleyi and B. sudanica were present at Lake Albert, while the two morphotypes (non-lacustrine and lacustrine) of B. choanomphala were present at Lake Victoria. In addition, an invasive, unidentified Asian Gyraulus species was present at Lake Albert and Lake Victoria. The shells are viewed from the apertural (left) and umbilical (right) shell angles. (TIF) [file pntd.0011506.s002.tif]

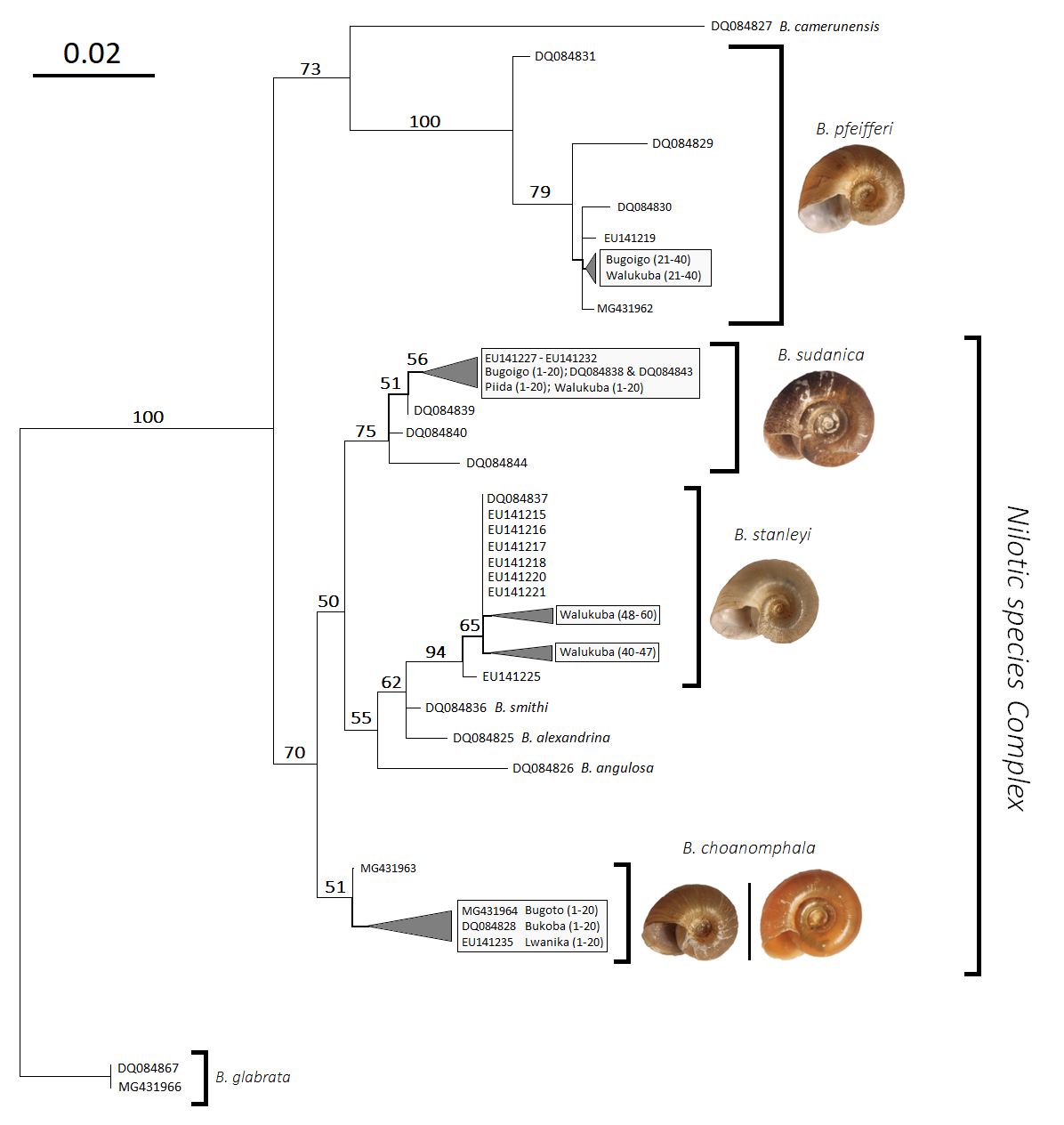

Supplement: S3 Fig — This tree was generated using PhyML v3.1 using a GTR+Γ model and is rooted on Biomphalaria glabrata. Numbers on branches indicate the bootstrap percentages for 1000 replicates (bootstrap support values under 50% are not shown). The scale bar represents sequence divergence. Samples labelled ‘cf.’ had shell morphologies’ that looked like a specific species but were identified by the original authors as another species using molecular methods. (TIF) [file pntd.0011506.s003.tif]

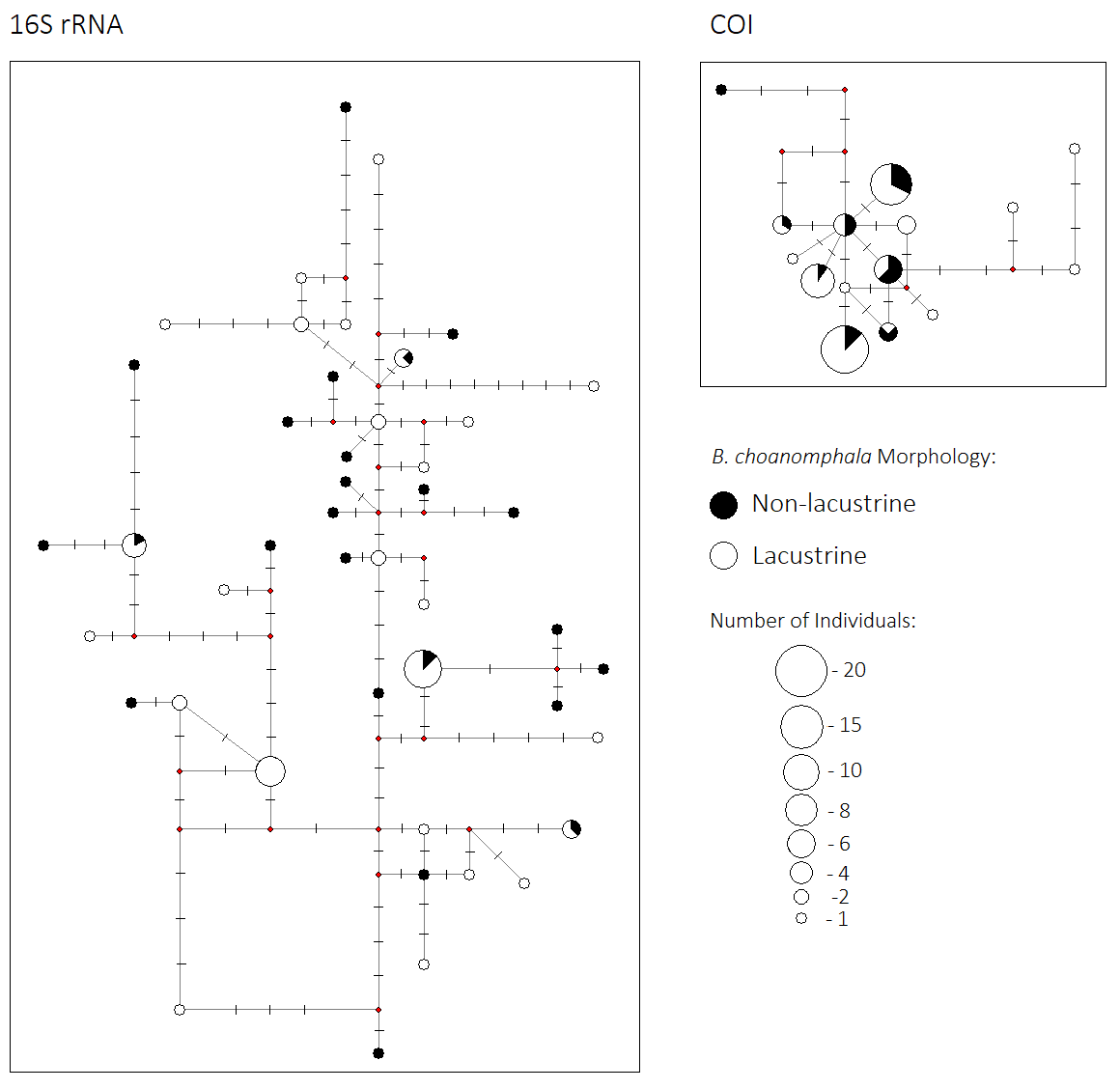

Supplement: S4 Fig — Each of the B. choanomphala snails shown are colour-coded to indicate whether they exhibited a non- lacustrine (black) or lacustrine (white) shell morphology. This network was generated using the software NETWORK v5. Circles represent each haplotype and circle size represents the numbers of individuals sharing a haplotype. Diamonds represent intermediate haplotypes, while hatch marks between points represent the number of nucleotide substitutions (substitutions more than five are indicated by numbers). Gaps were included in the 16S and COI alignments. (TIF) [file pntd.0011506.s004.tif]

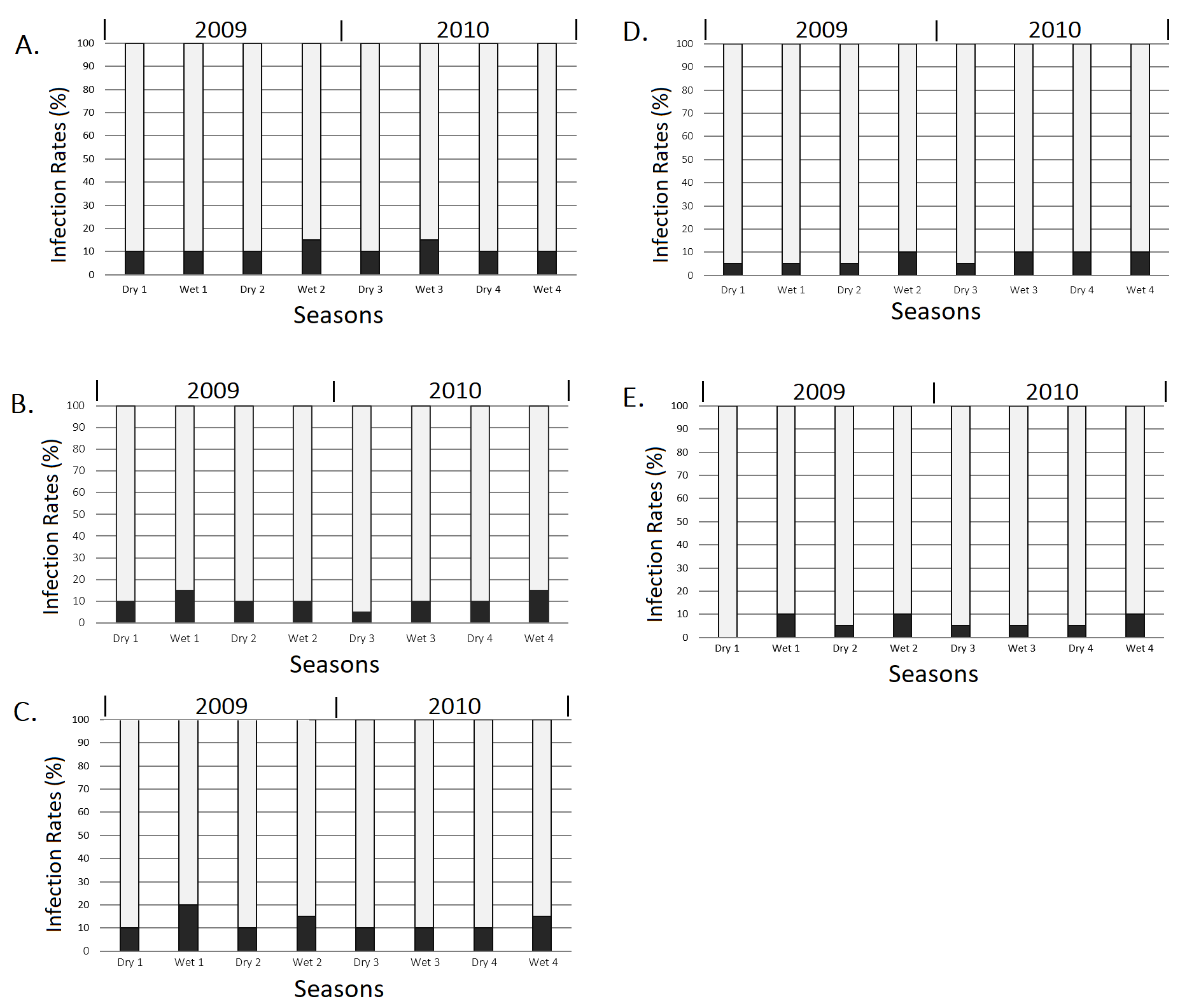

Supplement: S5 Fig — Seasonal prevalence of Schistosoma mansoni infection at our Lake Albert (A-C) and Lake Victoria (D-E) sites over the course of two years (2009–2010). Biomphalaria sudanica (n = 320) was tested at two sites in Lake Albert (A: Bugoigo & B: Walukuba), while B. pfeifferi (n = 160) was tested at one site (C: Walukuba). Biomphalaria choanomphala (n = 320) was tested at two sites at Lake Victoria (D: Bugoto & E: Lwanika). Black bars indicate the percentage of infected individuals (n = 20). (Dry 1: January-February 2009; Wet 1: March-May 2009; Dry 2: June-August 2009; Wet 2: September-November 2009; Dry 3: December 2009-February 2010; Wet 3: March-May 2010; Dry 4: June-August 2010; Wet 4: September-November 2010). (TIF) [file pntd.0011506.s005.tif]
