# Supplementary material for: Seasonal patterns of Schistosoma mansoni infection within Biomphalaria snails at the Ugandan shorelines of Lake Albert and Lake Victoria
Source: PLoS Negl Trop Dis. 2023 Aug 14;17(8):e0011506. doi: 10.1371/journal.pntd.0011506 (PMC10424865; doi:10.1371/journal.pntd.0011506)
Supplement: S1 Table — (DOCX) [file pntd.0011506.s006.docx]

S1 Table. GenBank accession numbers and corresponding references for the 16S/COI phylogenetic tree.

|  | Accession no. | | Species | Reference |
| --- | --- | --- | --- | --- |
|  | 16S rRNA | COI |  |  |
| BsmRwe1 | AY577474 | DQ084836 | *smithi* | Jørgensen et al. (2007) [1] |
| BalDBL1 | DQ084847 | DQ084825 | *alexandrina* | Jørgensen et al. (2007) [1] |
| BanRua1 | DQ084848 | DQ084826 | *angulosa* | Jørgensen et al. (2007) [1] |
| BcaBak1 | DQ084849 | DQ084827 | *camerunensis* | Jørgensen et al. (2007) [1] |
| BchVic1 | DQ084850 | DQ084828 | *choanomphala* | Jørgensen et al. (2007) [1] |
| BpfChi1 | DQ084851 | DQ084829 | *pfeifferi* | Jørgensen et al. (2007) [1] |
| BpfKib1 | DQ084852 | DQ084830 | *pfeifferi* | Jørgensen et al. (2007) [1] |
| BpfDeG1 | DQ084853 | DQ084831 | *pfeifferi* | Jørgensen et al. (2007) [1] |
| BstBut1 | DQ084858 | DQ084837 | *stanleyi* | Jørgensen et al. (2007) [1] |
| BsuBut1 | DQ084859 | DQ084838 | *sudanica* | Jørgensen et al. (2007) [1] |
| BsuKin1 | DQ084860 | DQ084839 | *sudanica* | Jørgensen et al. (2007) [1] |
| BsuMah1 | DQ084861 | DQ084840 | *sudanica* | Jørgensen et al. (2007) [1] |
| BsuNto1 | DQ084864 | DQ084843 | *sudanica* | Jørgensen et al. (2007) [1] |
| BsuRut1 | DQ084865 | DQ084844 | *sudanica* | Jørgensen et al. (2007) [1] |
| FL1 | EU141175 | EU141215 | *stanleyi* | Plam et al. (2008) [2] |
| FL2 | EU141176 | EU141216 | *stanleyi* | Plam et al. (2008) [2] |
| FL3 | EU141177 | EU141217 | *stanleyi* | Plam et al. (2008) [2] |
| FL4 | EU141178 | EU141218 | *stanleyi* | Plam et al. (2008) [2] |
| FL5 | EU141179 | EU141219 | *pfeifferi* | Plam et al. (2008) [2] |
| FL6 | EU141180 | EU141220 | *stanleyi* | Plam et al. (2008) [2] |
| FN1 | EU141181 | EU141221 | *stanleyi* | Plam et al. (2008) [2] |
| FN5 | EU141185 | EU141225 | *stanleyi* | Plam et al. (2008) [2] |
| SN1 | EU141187 | EU141227 | *sudanica* | Plam et al. (2008) [2] |
| SN2 | EU141188 | EU141228 | *sudanica* | Plam et al. (2008) [2] |
| SN3 | EU141189 | EU141229 | *sudanica* | Plam et al. (2008) [2] |
| SN4 | EU141190 | EU141230 | *sudanica* | Plam et al. (2008) [2] |
| SN5 | EU141191 | EU141231 | *sudanica* | Plam et al. (2008) [2] |
| SN6 | EU141192 | EU141232 | *sudanica* | Plam et al. (2008) [2] |
| - | MG431962 | MG431962 | *pfeifferi* | Zhang et al. (2018) [3] |
| - | MG431963 | MG431963 | *cf. sudanica* | Zhang et al. (2018) [3] |
| - | MG431964 | MG431964 | *choanomphala* | Zhang et al. (2018) [3] |
| - | MG431966 | MG431966 | *glabrata* | Zhang et al. (2018) [3] |
|  | HM768950 | HM769133 | *choanomphala* | Standley et al. (2014) [4] |

Note: ‘cf.’ indicates the shell morphology looked like a specific species but was identified as a different species by the original authors using molecular methods.

**References:**

1. Jørgensen A, Kristensen TK, Stothard JR. Phylogeny and biogeography of African Biomphalaria (Gastropoda: Planorbidae), with emphasis on endemic species of the great East African lakes. Zoological Journal of the Linnean Society. 2007;151(2):337-349.
2. Plam M, Jørgensen A, Kristensen TK, Madsen H. Sympatric Biomphalaria species (Gastropoda: Planorbidae) in Lake Albert, Uganda, show homoplasies in shell morphology. African Zoology. 2008;43(1):34-44.
3. Zhang SM, Bu L, Laidemitt MR, Lu L, Mutuku MW, Mkoji GM, et al. Complete mitochondrial and rDNA complex sequences of important vector species of Biomphalaria, obligatory hosts of the human-infecting blood fluke, Schistosoma mansoni. Scientific reports. 2018;8(1):7341.
4. Standley CJ, Goodacre SL, Wade CM, Stothard JR. The population genetic structure of Biomphalaria choanomphala in Lake Victoria, East Africa: implications for schistosomiasis transmission. Parasites & vectors. 2014;7:1-0.
