# Supplementary material for: Seasonal patterns of Schistosoma mansoni infection within Biomphalaria snails at the Ugandan shorelines of Lake Albert and Lake Victoria
Source: PLoS Negl Trop Dis. 2023 Aug 14;17(8):e0011506. doi: 10.1371/journal.pntd.0011506 (PMC10424865; doi:10.1371/journal.pntd.0011506)
Supplement: S2 Table — (DOCX) [file pntd.0011506.s007.docx]

S2 Table. GenBank accession numbers for the 16S and COI haplotype network.

| 16S | | |
| --- | --- | --- |
|  | Accession no. | Species |
| FN2 | EU141182 | *cf. choanomphala* |
| - | AY030202 | *choanomphala* |
| H44 | HM768993 | *choanomphala* |
| H60 | HM769009 | *choanomphala* |
| H68 | HM769017 | *choanomphala* |
| H80 | HM769029 | *choanomphala* |
| H84 | HM769033 | *choanomphala* |
| H85 | HM769034 | *choanomphala* |
| H86 | HM769035 | *choanomphala* |
| H101 | HM769050 | *choanomphala* |
| H137 | HM769086 | *choanomphala* |
| H145 | HM769094 | *choanomphala* |
| H147 | HM769096 | *choanomphala* |
| H150 | HM769099 | *choanomphala* |
| H160 | HM769109 | *choanomphala* |
| - | MG431964 | *choanomphala* |
| AJ-2005 | DQ084854 | *cf. pfeifferi* |
| FL5 | EU141179 | *cf. pfeifferi* |
| LS-131 | AY030193 | *pfeifferi* |
| LS-135 | AY030194 | *pfeifferi* |
| LS-82 | AY030195 | *pfeifferi* |
| LS-336 | AY030196 | *pfeifferi* |
| - | AY126599 | *pfeifferi* |
| - | AY126600 | *pfeifferi* |
| - | AY126601 | *pfeifferi* |
| - | AY126602 | *pfeifferi* |
| - | AY126603 | *pfeifferi* |
| - | AY126604 | *pfeifferi* |
| - | AY126605 | *pfeifferi* |
| - | AY126606 | *pfeifferi* |
| 1126 | AY198048 | *pfeifferi* |
| 1132 | AY198049 | *pfeifferi* |
| 1133 | AY198050 | *pfeifferi* |
| 1136 | AY198051 | *pfeifferi* |
| 115 | AY198052 | *pfeifferi* |
| 116 | AY198053 | *pfeifferi* |
| 117 | AY198054 | *pfeifferi* |
| 131 | AY198055 | *pfeifferi* |
| 133 | AY198056 | *pfeifferi* |
| 135 | AY198057 | *pfeifferi* |
| 137 | AY198058 | *pfeifferi* |
| 2004 | AY198059 | *pfeifferi* |
| 2005a | AY198060 | *pfeifferi* |
| 2098 | AY198061 | *pfeifferi* |
| 2100 | AY198062 | *pfeifferi* |
| 2005b | AY198063 | *pfeifferi* |
| 228a | AY198064 | *pfeifferi* |
| 262 | AY198065 | *pfeifferi* |
| 271 | AY198066 | *pfeifferi* |
| 274 | AY198067 | *pfeifferi* |
| 296 | AY198068 | *pfeifferi* |
| 311 | AY198069 | *pfeifferi* |
| 336 | AY198070 | *pfeifferi* |
| 337 | AY198071 | *pfeifferi* |
| 343 | AY198072 | *pfeifferi* |
| 350 | AY198073 | *pfeifferi* |
| 351 | AY198074 | *pfeifferi* |
| 82 | AY198075 | *pfeifferi* |
| 83 | AY198076 | *pfeifferi* |
| 92 | AY198077 | *pfeifferi* |
| - | AY577475 | *pfeifferi* |
| BpfChi1 | DQ084851 | *pfeifferi* |
| BpfKib1 | DQ084852 | *pfeifferi* |
| BpfDeG1 | DQ084853 | *pfeifferi* |
| BpfAbu1 | DQ084857 | *pfeifferi* |
| - | MG431962 | *pfeifferi* |
| - | AY030197 | *cf. stanleyi* |
| BstBut1 | DQ084858 | *stanleyi* |
| FL1 | EU141175 | *stanleyi* |
| FL2 | EU141176 | *stanleyi* |
| FL3 | EU141177 | *stanleyi* |
| FL4 | EU141178 | *stanleyi* |
| FL6 | EU141180 | *stanleyi* |
| FN1 | EU141181 | *stanleyi* |
| FN5 | EU141185 | *stanleyi* |
| - | AY126608 | *cf. sudanica* |
| - | MG431963 | *cf. sudanica* |
| BsuBut1 | DQ084859 | *sudanica* |
| BsuKin1 | DQ084860 | *sudanica* |
| BsuMah1 | DQ084861 | *sudanica* |
| BsuNto1 | DQ084864 | *sudanica* |
| BsuRut1 | DQ084865 | *sudanica* |
| SN1 | EU141187 | *sudanica* |
| SN2 | EU141188 | *sudanica* |
| SN3 | EU141189 | *sudanica* |
| SN4 | EU141190 | *sudanica* |
| SN5 | EU141191 | *sudanica* |
| SN6 | EU141192 | *sudanica* |

| COI | | |
| --- | --- | --- |
|  | Accession no. | Species |
| - | MG431964 | *choanomphala* |
| BchVic1 | DQ084828 | *choanomphala* |
| - | OL423116 | *pfeifferi* |
| - | MG431962 | *pfeifferi* |
| 1792 | AF199097 | *pfeifferi* |
| 1869 | AF199102 | *pfeifferi* |
| 1903 | AF199100 | *pfeifferi* |
| 1907 | AF199101 | *pfeifferi* |
| 1914 | AF199104 | *pfeifferi* |
| 1915 | AF199099 | *pfeifferi* |
| BpfChi1 | DQ084829 | *pfeifferi* |
| BpfDeG1 | DQ084831 | *pfeifferi* |
| BpfKib1 | DQ084830 | *pfeifferi* |
| MP05Bi1 | OM535896 | *pfeifferi* |
| MP05Bi2 | OM535897 | *pfeifferi* |
| SUDAN 0 | MG780151 | *pfeifferi* |
| SUDAN 22 | MG780154 | *pfeifferi* |
| SUDAN 23 | MG780155 | *pfeifferi* |
| SUDAN 24 | MG780156 | *pfeifferi* |
| SUDAN 26 | MG780157 | *pfeifferi* |
| SUDAN 27 | MG780158 | *pfeifferi* |
| SUDAN 28 | MG780160 | *pfeifferi* |
| SUDAN 29 | MG780161 | *pfeifferi* |
| SUDAN 3 | MG780150 | *pfeifferi* |
| SUDAN 31 | MG780159 | *pfeifferi* |
| SUDAN 32 | MG780162 | *pfeifferi* |
| SUDAN 33 | MG780163 | *pfeifferi* |
| SUDAN 34 | MG780164 | *pfeifferi* |
| SUDAN 35 | MG780165 | *pfeifferi* |
| SUDAN 36 | MG780166 | *pfeifferi* |
| SUDAN 37 | MG780167 | *pfeifferi* |
| SUDAN 38 | MG780168 | *pfeifferi* |
| SUDAN 40 | MG780174 | *pfeifferi* |
| SUDAN 41 | MG780175 | *pfeifferi* |
| SUDAN 42 | MG780176 | *pfeifferi* |
| SUDAN 43 | MG780177 | *pfeifferi* |
| SUDAN 44 | MG780178 | *pfeifferi* |
| SUDAN 45 | MG780179 | *pfeifferi* |
| SUDAN 7 | MG780152 | *pfeifferi* |
| SUDAN 8 | MG780153 | *pfeifferi* |
| SUDAN S13 | MG780170 | *pfeifferi* |
| SUDAN S15 | MG780169 | *pfeifferi* |
| SUDAN S20 | MG780171 | *pfeifferi* |
| SUDAN S21 | MG780172 | *pfeifferi* |
| SUDAN S25 | MG780173 | *pfeifferi* |
| ZWE 2 | MG780180 | *pfeifferi* |
| ZWE 232 | MG780207 | *pfeifferi* |
| ZWE 236 | MG780208 | *pfeifferi* |
| ZWE 254 | MG780197 | *pfeifferi* |
| ZWE 258 | MG780196 | *pfeifferi* |
| ZWE 260 | MG780195 | *pfeifferi* |
| ZWE 261 | MG780194 | *pfeifferi* |
| ZWE 262 | MG780193 | *pfeifferi* |
| ZWE 263 | MG780192 | *pfeifferi* |
| ZWE 264 | MG780191 | *pfeifferi* |
| ZWE 266 | MG780190 | *pfeifferi* |
| ZWE 267 | MG780189 | *pfeifferi* |
| ZWE 268 | MG780188 | *pfeifferi* |
| ZWE 269 | MG780187 | *pfeifferi* |
| ZWE 27 | MG780198 | *pfeifferi* |
| ZWE 270 | MG780186 | *pfeifferi* |
| ZWE 271 | MG780185 | *pfeifferi* |
| ZWE 276 | MG780184 | *pfeifferi* |
| ZWE 277 | MG780183 | *pfeifferi* |
| ZWE 278 | MG780182 | *pfeifferi* |
| ZWE 280 | MG780181 | *pfeifferi* |
| ZWE 30 | MG780202 | *pfeifferi* |
| ZWE 32 | MG780203 | *pfeifferi* |
| ZWE 33 | MG780199 | *pfeifferi* |
| ZWE 33 | MG780204 | *pfeifferi* |
| ZWE 37 | MG780200 | *pfeifferi* |
| ZWE 37 | MG780201 | *pfeifferi* |
| ZWE 38 | MG780205 | *pfeifferi* |
| ZWE 41 | MG780206 | *pfeifferi* |
| BstBut1 | DQ084837 | *stanleyi* |
| - | OL423117 | *cf. sudanica* |
| - | MG431963 | *cf. sudanica* |
| 1986 | AF199106 | *cf. sudanica* |
| 1987 | AF199107 | *cf. sudanica* |
| 1091 | AF199088 | *sudanica* |
| 1908 | AF199087 | *sudanica* |
| 1925 | AF199108 | *sudanica* |
| BsuBut1 | DQ084838 | *sudanica* |
| BsuKin1 | DQ084839 | *sudanica* |
| BsuMah1 | DQ084840 | *sudanica* |
| BsuNto1 | DQ084843 | *sudanica* |
| BsuRut1 | DQ084844 | *sudanica* |

Note: ‘cf.’ indicates the shell morphology looked like a specific species but was identified as a different species by the original authors using molecular methods.
