# Supplementary material for: Seasonal patterns of Schistosoma mansoni infection within Biomphalaria snails at the Ugandan shorelines of Lake Albert and Lake Victoria
Source: PLoS Negl Trop Dis. 2023 Aug 14;17(8):e0011506. doi: 10.1371/journal.pntd.0011506 (PMC10424865; doi:10.1371/journal.pntd.0011506)
Supplement: S3 Table — (DOCX) [file pntd.0011506.s008.docx]

S3 Table. Infection prevalence of the single time point dataset and the seasonality dataset for our Lake Albert and Lake Victoria sites.

| Lake Albert | | | | | | | |
| --- | --- | --- | --- | --- | --- | --- | --- |
| Site | Samples Preserved | Species | Samples Tested | | Number Infected | | Total |
|  |  |  | D1 | D2 |  |  |  |
| Bugoigo | 977 | *B. pfeifferi* | *n=*20 | *-* | 10% | (2/20) | 11.7% (70/600) |
|  |  | *B. sudanica* | *n=*20 | *n=*160 | 11.7% | (21/180) |  |
| Piida | 521 | *B. sudanica* | *n=*20 | *-* | 10% | (2/20) |  |
| Walukuba | 1147 | *B. pfeifferi* | *n=*20 | *n=*160 | 12.2% | (22/180) |  |
|  |  | *B. stanleyi* | *n=*20 | *-* | 15% | (3/20) |  |
|  |  | *B. sudanica* | *n=*20 | *n=*160 | 11.1% | (20/180) |  |
| Lake Victoria | | | | | | | |
| Site | Samples Preserved | Species | Samples Tested | | Number Infected | | Total |
|  |  |  | D1 | D2 |  |  |  |
| Bugoto | 4005 | *B. choanomphala* | *n=*20 | *n=*160 | 6.1% | (11/180) | 6.8% (26/380) |
| Bukoba | 1264 | *B. choanomphala* | *n=*20 | *-* | 5% | (1/20) |  |
| Lwanika | 1113 | *B. choanomphala* | *n=*20 | *n=*160 | 7.8% | (14/180) |  |

Note: D = Dataset. D1 was the single time point dataset and D2 was the seasonality dataset.
